# A New MBH Adduct as an Efficient Ligand in the Synthesis of Metallodrugs: Characterization, Geometrical Optimization, XRD, Biological Activities, and Molecular Docking Studies

**DOI:** 10.3390/molecules27238150

**Published:** 2022-11-23

**Authors:** Shazia Ishfaq, Shazia Nisar, Sadaf Iqbal, Saqib Ali, Syed Tariq Ali, ElSayed Din, Norah Salem Alsaiari, Kholood A. Dahlous, Muhammad Sufyan Javed, Patrizia Bocchetta

**Affiliations:** 1 Department of Chemistry, Faculty of Sciences, University of Karachi, Karachi 75270, Pakistan; 2 Department of Chemistry, Faculty of Basic and Applied Sciences, University of Kotli, Kotli 11100, Pakistan; 3 Faculty of Engineering and Technology, Future University in Egypt, New Cairo 11835, Egypt; 4 Department of Chemistry, College of Science, Princess Nourah bint Abdulrahman University, P.O. Box 84428, Riyadh 11671, Saudi Arabia; 5 Department of Chemistry, College of Science, King Saud University, Riyadh 11451, Saudi Arabia; 6 School of Physical Science and Technology, Lanzhou University, Lanzhou 730000, China; 7 Dipartimento di Ingegneria dell’Innovazione, Università del Salento, via Monteroni, 73100 Lecce, Italy

**Keywords:** MBH adduct, metal complexes, XRD, biological studies, geometrical optimization

## Abstract

This article reports the synthesis, characterization, geometrical optimization, and biological studies of new MBH-based organometallic compounds of medicinal significance. The ligand (MNHA) was prepared via the Morita–Baylis–Hillman (MBH) synthetic route, from aromatic aldehyde containing multiple functional groups. Metal complexes were prepared in an alkaline medium and under other suitable reaction conditions. Spectral and elemental analyses were used to identify the structural and molecular formulas of each compound. Optimized geometry was determined through density functional theory (DFT) B3LYP and 6-311++ G (d,p) basis set for the MBH adduct, whereas structures of novel complexes were optimized with the semi-empirical PM6 method. Powder XRD analysis furnished the crystal class of complexes, with Co^3+^, Cr^3+^, and Mn^2+^ being cubic, while Ni^2+^ was hexagonal, and Cu^2+^ was orthorhombic. Moreover, the ligand, along with Ni^2+^ and Co^3+^ complexes, showed profound antibacterial action against *S. aureus*, *E. coli*, *B. pumilis*, and *S. typhi*. Additionally, all of the complexes were shown to persist in the positive antioxidant potential of the ligand. Contrarily, not a single metal complex conserved the antifungal potentials of the ligand.

## 1. Introduction

The MBH reaction is one of the major themes of multipurpose and significant practice in most organic preparations [1]; it possesses remarkable applications as an essential initiative [2,3] as well. The adducts prepared through MBH methodology are extremely multifunctional, especially the allylic alcohols and esters, which have been verified to be important building blocks for natural products and biologically vital compounds [4]. As a consequence of having polar moieties in their structures, they are very significant in homo- or heterocyclic syntheses with multiple functionalities [5]. The MBH reaction is valuable to the atom economy, which can produce extraordinary, medicinally active drugs. It involves a reaction of C-C bond formation between sp^2^ carbons of an aldehyde as well as an electron-deficient unsaturated hydrocarbon. Under specific reaction conditions, and by using nucleophilic amines or phosphines as catalysts, a powerful multifunctional adduct is achieved [6,7]. Moreover, in the presence of more than one similar position, C-C bond construction at the selective site of the requisite compound is another major challenge for chemists [8]. The alkyl group in the selective aldehyde mostly comprises an aromatic ring with dissimilar functional groups attached to it. The functional groups, such as -OH, C=O, -NO_2_, and C=C, not only provide the binding sites, but their different positions on the aromatic ring also enhance their functionality [9,10]. 

Owing to the availability of different sites, our focus was to prepare the MBH adduct methyl 2-((2-nitrophenyl)(hydroxy)methyl) acrylate (MNHA) as a product with minimum torsion strain. Afterward, this active adduct was subjected to coordinate as a ligand to the central transition metal ions. The binding of an aromatic aldehyde with an activated alkene introduces another functional group into the same structure at the point of junction with the adduct [11]. In addition, the -OH functional group is also very important due to its ease of participation in coordination towards central transition metal ions after de-protonation [12]. Furthermore, the selected activated alkene, methyl acrylate, has oxygen moieties of functional groups along with the 𝛑-bond. These oxygen-containing functional groups (C=O, ROCH_3_) are extremely attractive in nature, and function as nucleophiles to the Lewis acid (transition metal) due to the presence of lone pairs of electrons [13]. Additionally, the compounds that contain NO_2_ functional groups on the aromatic ring function as substrates with an adoptable nature, because these compounds can easily undergo diverse and useful synthetic conversions, e.g., transformation, substitution, and addition. Compounds that contain the NO_2_ group, particularly nitrobenzaldehydes, are a major class of compounds that have been extensively investigated for their biological assays [8,14]. The biological significance of this class of compounds, which sometimes function as intermediates in the preparation of numerous powerful medications and natural products, marks them as creationistic compounds in the medicinal domain [15]. When these adduct-type species coordinate with metal ions, which already have contributed to many biological functions, for example, antibacterial, leishmanicidal, antifungal, antioxidant, and anticancer actions [16,17,18], they can provoke these capabilities. In addition, it is more interesting to prepare a novel drug with such pharmaceutically significant compounds. Recently, many MBH adducts have been used in various pathogenic disorders. Therefore, MBH adducts are widely utilized as the primary material of versatile synthetic drugs [19]. 

Our main objective to pursue this study was to synthesize transition metal complexes from a pharmaceutically important MBH adduct. To provide a greater affinity to coordinate the central metal ion [20,21,22], the selective reactants as starting material with little modification were used. Although both MBH adduct (MNHA) and transition metals could act as active drugs against most of the pathogenic strains, we synthesized metal complexes with the consideration that they might bear a greater potential than the ligand and the metals themselves. The structural confirmation of MNHA was achieved via spectral (UV-Visible, FT-IR, ^1^H NMR, elemental, and mass spectroscopy) analysis, as well as DFT calculations [23]. Some of these compounds were found to be proficient in fighting against microbial pathogens. They all possess antioxidant potential, but antifungal activity is exhibited by MNHA only. Our continuous struggle in the investigation of biologically active MBH adducts inspired us to synthesize their metal complexes, which could act as better and improved drugs.

## 2. Experimental

### Materials and Methods

In the present study, all of the solvents, such as ethyl acetate, n-hexane, and methanol of synthetic grade, were used without further purification. Precautionary measures were taken to avoid the contamination of oxygen and moisture during the synthesis of the compounds. Furthermore, the reagents 2-Nitrobenzaldehyde, 1,4-diazabicyclo[2.2.2]octane (DABCO), CuCl_2_·2H_2_O, MnCl_2_·2H_2_O, NiCl_2_·6H_2_O, CoCl_3_·6H_2_O, and CrCl_3_·6H_2_O were purchased from Sigma-Aldrich. Methyl acrylate, NaCl, MgSO_4_, and NaOMe, of Dae-Jung Kosdaq Reagents & Chemicals, were employed. Thin-layer chromatography was carried out using TLC cards that were coated with silica gel, which were monitored under a UV lamp. A digital hot plate with a constant temperature and stirring system was used during synthesis. The electronic spectra of ligands and complexes were recorded with a LAMBDA 1050+ UV/Vis/NIR spectrophotometer in DMSO solvent at concentrations around 1 × 10^−3^ M. A Gallen Kamp melting point instrument was used to record the melting points of synthesized compounds **3–8**. A Fourier transform infrared (FT-IR) instrument prestige-21 distributed by Shimadzu Company was used to record FT-IR spectra of compound 3 and its respective metal complexes (**4–8**) in the spectral range of 4000–500 cm^−1^, in anhydrous pallets of KBr. The ^1^H NMR spectrum of the ligand was recorded on an AVANCE NEO NMR spectrometer in CDCl_3_ solvent (500 MHz for ^1^H). Chemical shifts (δ) and coupling constants (*J*) were reported in ppm and Hz units, respectively, keeping TMS as an internal reference. A JEOL 600H-1 mass spectrophotometer instrument by Direct Probe Inlet at ionization mode EI+ was used to obtain mass spectra for the compounds, and data were expressed in the form of spectra. The resulting mass spectra were presented typically as a function of mass-to-charge (*m/z*) ratio versus relative abundance (%). Elemental analyses performed on a LECO CHNS 932 model micro-analytical instrument showed an agreement better than ±0.3% with the calculated values. The XRD study (powder pattern) of the complexes was carried out with the help of an X-ray diffractometer (Goniometer Radius mm = 240.00, Dist. Focus-Diverg. Slit mm = 91.00) with Cu-anode material, Kα [Å] = 1.54060, and generator settings 30 mA, 40 kV. Pananalytical company X-pro serial No. DYH313 was the major instrument used for data analysis.

## 3. Synthesis

### 3.1. Synthesis of Methyl 2-((2-nitrophenyl)(hydroxy)methyl) Acrylate (MNHA)

**Scheme 1:**
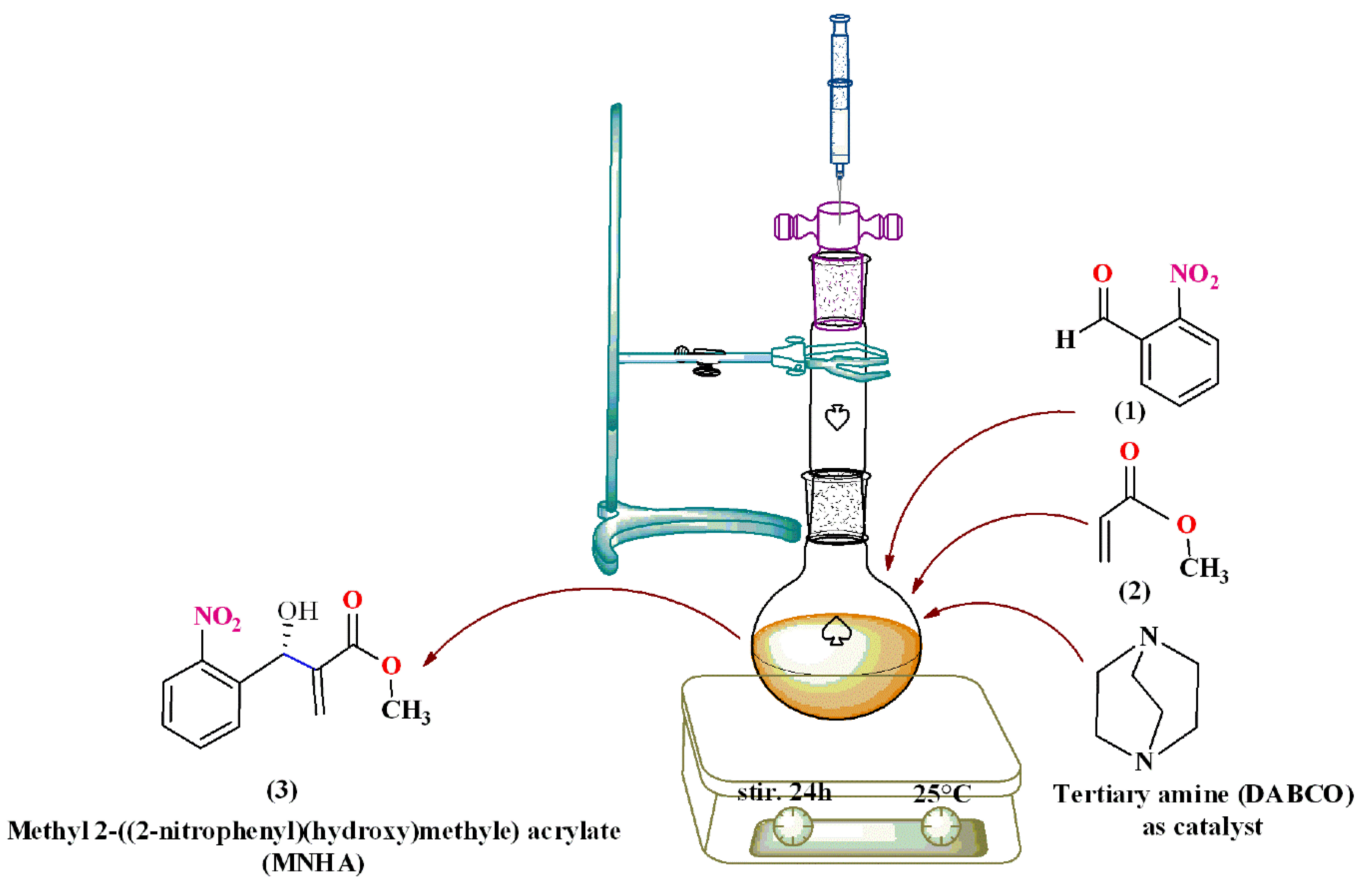
General scheme of Morita–Baylis–Hillman reaction with tertiary amine as a catalyst at room temperature and other reaction conditions.

The synthesis of MNHA (compound 3) was initiated by the addition of 5 equivalents of methyl acrylate to 1 equivalent of 2-nitro benzaldehyde, in 5% THF as a solvent; 0.65 equivalents of DABCO (tertiary amine) as catalyst was also added [24,25]. The mixture in a round-bottomed flask was maintained under continuous stirring and room temperature; the reaction was found to be complete in about 24 h. TLC was run at regular intervals of one and a half hours, to check for completion of the reaction. After the evaporation of additional methyl acrylate, a viscous crude material was obtained as the end product. The crude was diluted by the addition of 15 mL of ethyl acetate and brine solution. A separating funnel was used to separate the polar and non-polar layers. To ensure the complete separation of organic and inorganic layers, the procedure was repeated three times. Moreover, through the evaporation of EtOAc, the organic phase was concentrated. The concentrated crude was run through column chromatography to obtain a product of maximum purification. The mobile phase system consisted of 5:95 to 35:65 (*v/v*) ethyl acetate and n-hexane. The required main spot was collected as a product in pure form, as confirmed through spectral analysis [7,26,27]. Scheme 1 represents the preparation of MNHA as a brownish-orange product, with a yield of 79.7% (237.06 g/mol); m.p = 124.3–126.5 °C; λ_max_ (nm): 300; ɛ_max_ (mol^−1^cm^−1^L): 1176.47. Mass spectrum [*m/z* (%)] = 237 (65) M^+^, 238 (3), 236 (2), 220 (71), 206 (6.1), 191 (25), 188 (100), 160 (30), 145 (35), 132 (32), 104 (15), 77(13) Elemental Anal. Calc. (%): C, 55.70; H, 4.67; N, 5.90; O, 33.72; found: C, 55.20; H, 4.56; N. 6.1; O, 32.98 for C_11_H_11_NO_5_. FT-IR (KBr, ῡ cm^−1^): 3477 (OH), 2958 (=C-H), 2875 (sp^3^ C-H stretch), 1720 (ester C=O), 1629 (aromatic C=C), 1529 and 1552 (-N=O), 1442 (-CH_3_), 1249 (C-O), 1149 (C-OCH_3_), 1045 (-OH bend), 744 (=C-H aromatic). ^1^H NMR (500 MHz, CDCl_3_): δ 3.419 (s, 1H, OH), 3.707 (s, 3H, OCH_3_), 5.705 (s, 1H, CH_2_), 6.175 (s, 1H, CH_2_), 6.342 (s, 1H, CH), 7.426 (t, 1H, *J*_2,3_ = 8.5 Hz, *J*_2,4_ =1.5 Hz H-2, Ar), 7.607 (t, 1H, *J*_3,2_ = 8.5 Hz, *J*_3,4_ = 7.5 Hz, H-3, Ar), 7.737 (d, 1H, *J*_4,5_ = 8 Hz, H-4, Ar), 7.915 (d, 1H*, J*_5,4_ = 8.0 Hz, H-5, Ar), ppm.

### 3.2. General Procedure for the Synthesis of MNHA Adduct-Based Metal Complexes (Compounds ***4***–***8***)

**Scheme 2:**
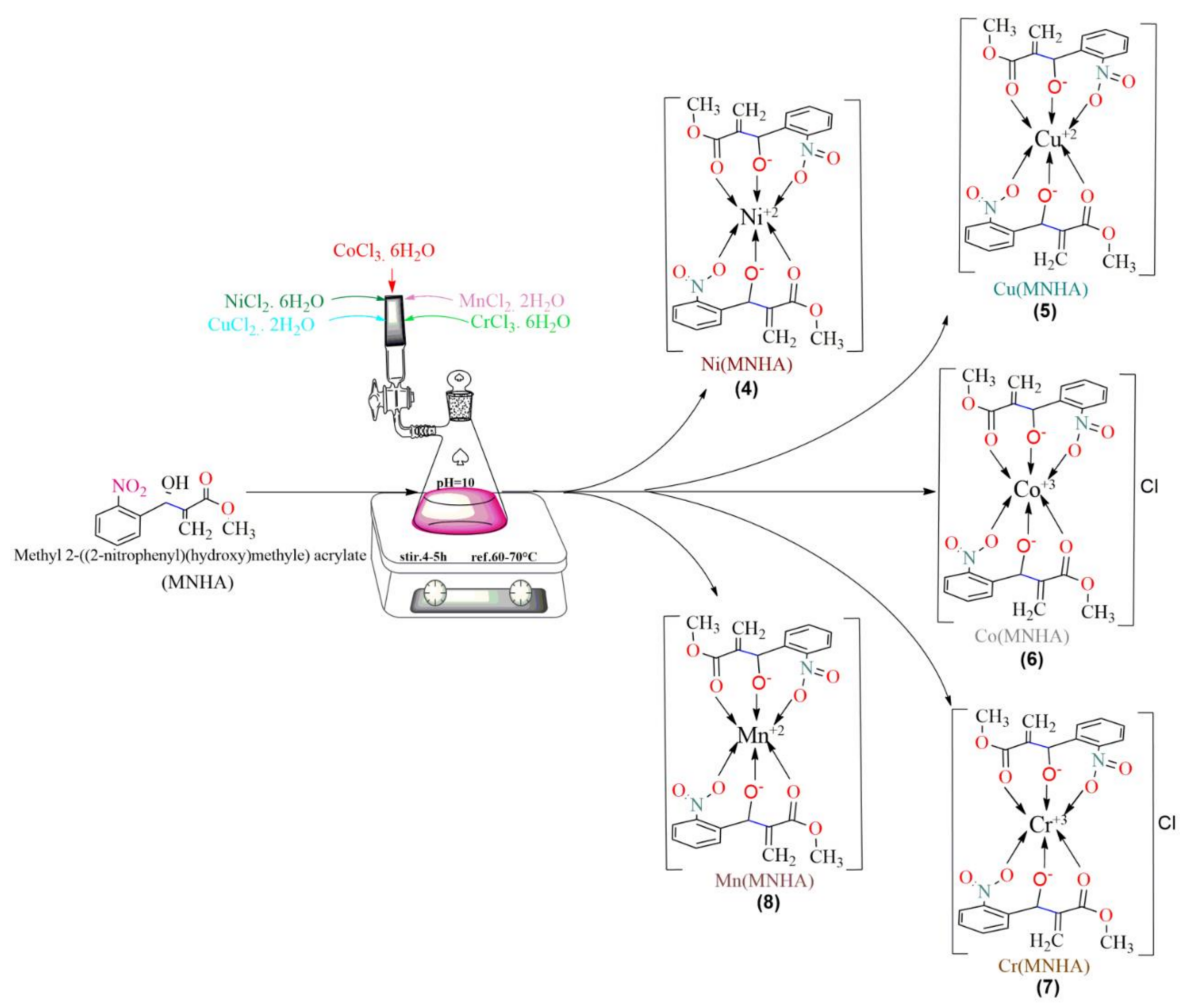
Methodology adapted for syntheses of compounds **4**–**8**.

There were 0.02 M Metal salt solutions in distilled water and 0.04 M solutions of MNHA ligand in hot ethanol (1:2) prepared. Solutions of 20 mL of metal salts (NiCl_2_·6H_2_O, CuCl_2_·2H_2_O, MnCl_2_·2H_2_O, CrCl_3_·6H_2_O, and CoCl_3_·6H_2_O) were mixed dropwise with the adduct solution. The mixture was maintained under constant stirring, and NaOH (2 M) was added to maintain the pH at 10. The mixture was refluxed at an optimized temperature of 60–70 °C for 4–5 h until a solid mass appeared [28]. The reaction mixture was cooled gently at room temperature, and the final product in the form of a precipitate was collected by means of vacuum filtration. The product was washed thrice successively with distilled water, and ethanol, and dried in a desiccator [12,29,30]. The methodology that was adapted for the synthesis of the metal complexes is shown in Scheme 2. The spectral analysis and molecular modeling helped to further deduce the composition of the synthesized metal complexes. As a result of the low solubility of the prepared complexes in polar and non-polar solvents, all of the attempts to grow single crystals failed. Therefore, to exactly determine the crystal class of each species, XRD powder diffraction was employed [30].

#### 3.2.1. [Ni(MNHA)_2_] (Compound **4**)

Penny brown color. Yield 86.7% (533.13 g/mol); m.p ≥ 300 °C; λ_max_ (nm): 370; ɛ_max_ (mol^−1^cm^−1^L): 74.35. Elemental Anal. Calc. (%): C, 49.56; H, 4.16; N, 5.25; O, 30.01; found: C, 50.20; H, 4.35; N. 4.9; O, 31.05 for [NiC_22_H_22_N_2_O_10_]^2+^. FT-IR (KBr, ῡ cm^−1^): 3450–3300 (OH), 2931 (=C-H), 2860 (sp^3^ C-H stretch), 1716 (ester C=O), 1610 (aromatic C=C), 1525 (-N=O), 1431 (-CH_3_), 1384 (C-O), 1141 (C-OCH_3_), 1111 (-OH bend), 651 (=C-H aromatic), 609 (M-O), 464 (M-NO_2_). ESI [M]^+^ *m/z* (estimated): [NiC_17_H_15_N_2_O_7_]^+^ = 417.77, [NiC_18_H_17_N_2_O_8_]^+^ = 447.78, [NiC_20_H_19_N_2_O_8_]^+^ = 473.81, [NiC_21_H_19_N_2_O_9_]^+^ = 501.80, [NiC_22_H_22_N_2_O_10_]^+^ = 533.81.

#### 3.2.2. [Cu(MNHA)_2_] (Compound **5**)

Shamrock green color. Yield 88.5% (537.96 g/mol); m.p ≥ 300 °C; λ_max_ (nm): 371; ɛ_max_ (mol^−1^cm^−1^L): 108.88. Elemental Anal. Calc. (%): C, 49.12; H, 4.12; Cu, 11.81; N, 5.21; O, 29.74; found: C, 50.12; H, 4.54; N, 5.38; O, 28.96 for [CuC_22_H_22_N_2_O_10_]^2+^. FT-IR (KBr, ῡ cm^−1^): 3480–3380 (OH), 2926 (=C-H), 2856 (sp^3^ C-H stretch), 1622 (aromatic C=C), 1388 (C-O), 1114 (C-OCH_3_), 607 (M-O), 472 (M-NO_2_). ESI [M]^+^ *m/z* (estimated): [CuC_17_H_15_N_2_O_7_]^+^ = 422.62, [CuC_18_H_17_N_2_O_8_]^+^ = 452.64, [CuC_20_H_19_N_2_O_8_]^+^ = 478.66, [CuC_21_H_19_N_2_O_9_]^+^ = 506.65, [CuC_22_H_22_N_2_O_10_]^+^ = 537.66.

#### 3.2.3. [Co(MNHA)_2_] (Compound **6**)

Dark gray color. Yield 82.87% (569.38 g/mol); m.p ≥ 300 °C; λ_max_ (nm): 371; ɛ_max_ (mol^−1^cm^−1^L): 115.238. Elemental Anal. Calc. (%): C, 46.41; H, 4.60; Co, 10.35; N, 4.92; O, 33.72; found: C, 46.54; H, 4.65; N, 5.03; O, 33.84 for [CoC_22_H_26_N_2_O_12_]^3+^. FT-IR (KBr, ῡ cm^−1^): 3450–3300 (OH), 2931 (=C-H), 2860 (sp^3^ C-H stretch), 1716 (ester C=O), 1610 (aromatic C=C), 1525 (-N=O), 1431 (-CH_3_), 1384 (C-O), 1141 (C-OCH_3_), 1111 (-OH bend), 651 (=C-H aromatic), 609 (M-O), 464 (M-NO_2_). ESI [M]^+^ *m/z* (estimated): [CoC_17_H_19_N_2_O_9_]^+^ = 454, [CoC_18_H_21_N_2_O_10_]^+^ = 484.03, [CoC_20_H_23_N_2_O_10_]^+^ = 510.08, [CoC_21_H_23_N_2_O_11_]^+^ = 538.05, [CoC_22_H_26_N_2_O_12_]^+^ = 569.16.

#### 3.2.4. [Cr(MNHA)_2_] (Compound **7**)

Caramel brown color. Yield 85.7% (533.13 g/mol); m.p ≥ 300 °C; λ_max_ (nm): 371; ɛ_max_ (mol^−1^cm^−1^L): 1408.16. Elemental Anal. Calc. (%): C, 46.98; H, 4.66; N, 4.98; O, 34.14; found: C, 46.89; H, 4.76; N, 5.07; O, 35.10 for [CrC_22_H_26_N_2_O_12_]^2+^. FT-IR (KBr, ῡ cm^−1^): 3500–3400 (OH), 2950 (=C-H), 2800 (sp^3^ C-H stretch), 1625 (aromatic C=C), 1527 (-N=O), 1390 (C-O), 1177 (C-OCH_3_), 1107 (-OH bend), 750 (=C-H aromatic), 603 (M-O), 842 (M-OH_2_), 514 (M-NO_2_). ESI [M]^+^ *m/z* (estimated): [CrC_17_H_19_N_2_O_9_]^+^ = 447.08, [CrC_18_H_21_N_2_O_10_]^+^ = 477.09, [CrC_20_H_23_N_2_O_10_]^+^ = 503.11, [CrC_21_H_23_N_2_O_11_]^+^ = 531.12, [CrC_22_H_26_N_2_O_12_]^+^ = 562.12.

#### 3.2.5. [Mn(MNHA)_2_] (Compound **8**)

Amber brown color. Yield 81.27% (565.38 g/mol); m.p ≥ 300 °C; λ_max_ (nm): 366; ɛ_max_ (mol^−1^cm^−1^L): 110.687. Elemental Anal. Calc. (%): C, 46.74; H, 4.64; Mn, 9.72; N, 4.95; O, 33.96; found: C, 46.65; H, 4.53; N, 4.97; O, 34.16 for [MnC_22_H_26_N_2_O_12_]^2+^. FT-IR (KBr, ῡ cm^−1^): 3450–3350 (OH), 2926 (=C-H), 2856 (sp^3^ C-H stretch), 1610 (aromatic C=C), 1560 (-N=O), 1388 (C-O), 1128 (C-OCH_3_), 611 (M-O), 750 (M-OH_2_), 509 (M-NO_2_). ESI [M]^+^ *m/z* (estimated): [MnC_17_H_19_N_2_O_9_]^+^ = 450.02, [MnC_18_H_21_N_2_O_10_]^+^ = 480.03, [MnC_20_H_23_N_2_O_10_]^+^ = 506.05, [MnC_21_H_23_N_2_O_11_]^+^ = 534.05, [MnC_22_H_26_N_2_O_12_]^+^ = 565.16.

### 3.3. Antibacterial Assay for Compounds ***3***–***8***

The agar disc diffusion method [31] facilitated the determination of antibacterial activity in all of the compounds [32,33]. Four bacterial strains including two Gram-negative (*Staphylococcus aureus, Escherichia coli*) and two Gram-positive (*Bacillus pumilis, Salmonella typhi*) were tested. The sterilization of extracts was carried out using a 0.45 µm pore size (millipore) sterile membrane syringe filter. A culture suspension of 1 mL of each strain with 25% transmittance at 530 nm was added to 100 mL of agar with antibiotic (No.11) at 45 °C. Moreover, about 25 mL of inoculated agar was transferred to each respective Petri dish (20 × 100 mm), which solidified and was marked (compounds **3**–**8**). Four holes, each 8 mm in diameter, were made using a sterile borer with an internal diameter of 6 mm, soon after complete solidification. A volume of 100 µL extract from each suspension was poured into its respective well and incubated for 24 h (37 °C). Under strict aseptic conditions, the whole experiment was performed in triplicate. The zone of inhibition (mm) after incubation was measured and presented in terms of % inhibition using Gentamycin (0.3%) as a standard [34,35,36].

### 3.4. DPPH Radical Scavenging Activity for Compounds ***3***–***8***

The DPPH (2,2-diphenyl-1-picrylhydrazyl) radical scavenging method was used for the determination of free radical scavenging activity [12,37] of the said compounds, because of its specificity for organometallic synthesis. A solution of DPPH (0.3 mM) was prepared in methanol, and 1 mL of this solution was mixed with each sample (10 mL) in varying concentrations (25 µg–100 µg). Incubation at 37 °C was conducted for half an hour. Furthermore, the absorbance was measured (515 nm) with reference to the methanol-treated control, and the percent radical scavenging activity was determined. Ascorbic acid was used as the standard [38].

### 3.5. Antifungal assay of Compounds ***3***–***8***

Antifungal activities of the ligand, MNHA, and its corresponding metal complexes (compounds **3**–**8**) were measured via the quantitative agar well diffusion assay [39] against *Aspergillus Niger, Aspergillus flavus, Aspergillus fumigatus*, and *Candida albicans*. A 1 mL solution of each compound was mixed with 100 mL of Sabouraud dextrose agar (SDA) (4.0%) and poured onto a plate. Wells (8 mm) were prepared upon complete solidification, and the test compound (100 µL) was poured into each. The plates were stood at room temperature for 30 min and further incubated at 25 °C for five days. After five days, the plates were checked and zones of inhibition were measured.

## 4. Results and Discussion

The current research began with the successful preparation of the Morita–Baylis–Hillman Adduct (MNHA), according to the methodology given in Scheme 1. A significant C-C bond was established between the aldehyde (compound **1**) and acrylate (compound **2**) at the given reaction conditions. The OH, NO_2_, and C=O functional groups from the adduct (compound **3**) were accountable to coordinate with the central transition metal ions Scheme 2. The synthesized products (compounds **4**–**8**) were isolated in amorphous form. Their elemental analyses were in good accordance with 1:2 metal to ligand ratios (compound **3**). It was found that the Cr^3+^, Mn^2+^, and Co^3+^ metals were also coordinated with two additional water molecules. Thus, the general molecular formulae [M(MNHA)_2_] [(M= Ni^2+^ (4), Cu^2+^ (5), Mn^2+^ (8)] and [M(MNHA)_2_]Cl [(M=Co^3+^ (6), Cr^3+^ (7),] were assigned to the products. All of the coordination compounds were air- stable, colored, and solid at room temperature. Furthermore, it was observed that the complexes (compounds **4**–**8**) were insoluble in water as well as other organic solvents, but only partially soluble in DMSO. The possible transition modes of electrons before and after coordination were examined with UV–visible spectroscopy in the range of 200–800 nm. The presence of water as moisture and other functional groups were confirmed further using FT-IR analysis. It was found that the MNHA functional groups’ frequencies were modified after coordination with the transition metals. Moreover, when more than one coordination site was present in an organic ligand, a basic medium was preferred for the formation of its respective metal complex [40,41,42]. The results of % analysis confirmed that there were no carbon-, hydrogen-, nitrogen-, and oxygen-containing compounds outside the coordination sphere as counter ions. The reason for this was that if they were the ions of the aforementioned elements, there would have been higher percentages of these elements than observed [40]. The ESI [M]^+^ *m/z* of MNHA (compound **3**) confirmed the exact mass of compound **3**. XRD powder diffraction suggested the exact crystal class, along with the crystal size of each of the prepared metal complexes. Additionally, computational investigations proved the optimized molecular geometry of each metal complex.

### 4.1. Physical Characteristics and Elemental Analysis

The ligand (MNHA) and corresponding metal complexes were analyzed using physical as well as elemental analyses (Table 1). It was observed that the ligand in pure form was a viscous liquid; meanwhile, the formed complexes were in an amorphous solid state. Moreover, elemental analysis strongly indicated that MNHA formed complexes with desired metal ions (Ni^2+^, Cu^2+^, Co^3+^, Cr^3+^, Mn^2+^), which conferred a molar ratio of 2:1. The results also suggested that all of the metal complexes were without any water molecules [36,43], (Table 1 and Scheme 2). These types of transition metal complexes have already been reported in the literature [44,45,46].

### 4.2. UV–Visible Spectroscopy

The resulting absorbances at the corresponding λ of the absorption bands in the compounds **3**–**8** are summarized in Table 2. The UV–visible region of compound **3** showed distinct bands at 250 and 266 nm due to 𝛑→𝛑* transitions. Additionally, it showed a small band at 275 nm and a strong band at 300 nm due to the **n→**𝛑* transition of the nitro functional group in the aromatic ring of the adduct (MNLA). These transitions (𝛑→𝛑* and **n→**𝛑*) were observed to be shifted comparatively towards higher absorptions upon the coordination with central metal ions. This change strongly supported that oxygen atoms of the esteric carbonyl group, hydroxo group, and nitro group from the adduct contributed to coordination. The complex of Ni^2+^ (compound **4**) showed bands at 264, 283, and 370 nm, which corresponded to the 𝛑→𝛑* and **n→**𝛑* transitions, respectively. This behavior of electronic transitions of the designed complexes has been well explained for the UV–visible region of octahedral, diamagnetic complexes [47]. Meanwhile, the transitions of Cu(MNLA) complex (5) exhibited protuberant bands at 266, 290, and 371 nm. Furthermore, the complex of cobalt (compound **6**) yielded two noticeable weak absorption bands at 268 nm and 308 nm in the UV spectrum, while a strong band was also observed at 371 nm. These transitions may be due to the 𝛑→𝛑* and **n→**𝛑* transitions [48]. The weak spectral bands that appeared in Cr(MNLA) (compound **7**) at 265 and 308 nm can be designated to the 𝛑→𝛑* transition in the complex, with the suggestion that the **n→**𝛑* transition was shifted to 371 nm [49,50]. Moreover, the transition bands in the complex of Mn^2+^ (compound **8**) were observed at 268 nm, 286 nm, and 366 nm, strictly approximating the paramagnetic and octahedral geometry because of the tridenticity of the ligand towards the central metal [51].

### 4.3. FT-IR Spectroscopy

**Figure 1:**
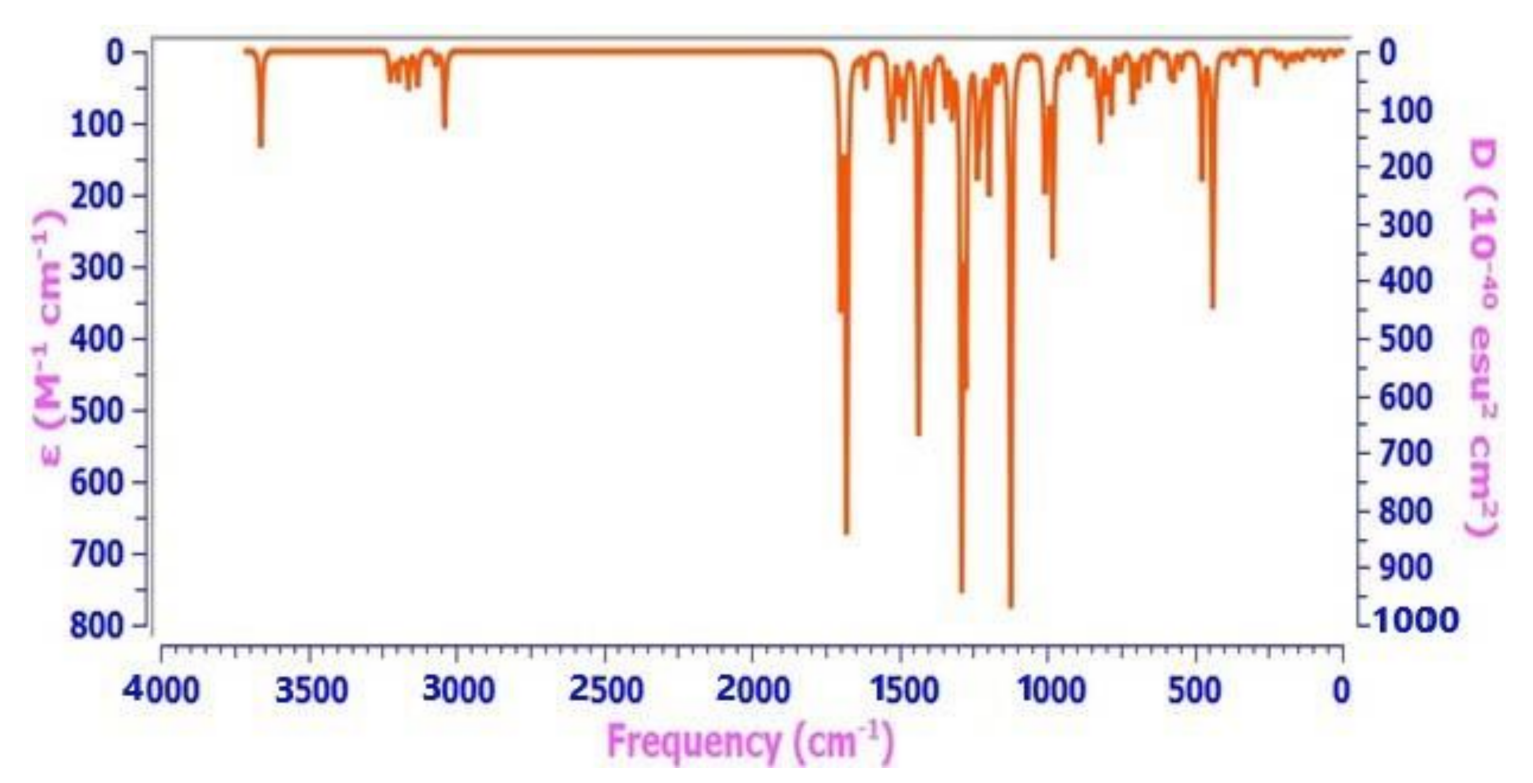
FT-IR theoretical spectrum of methyl 2-((2-nitrophenyl)(hydroxyl)methyl) acrylate (MNHA).

**Figure 2:**
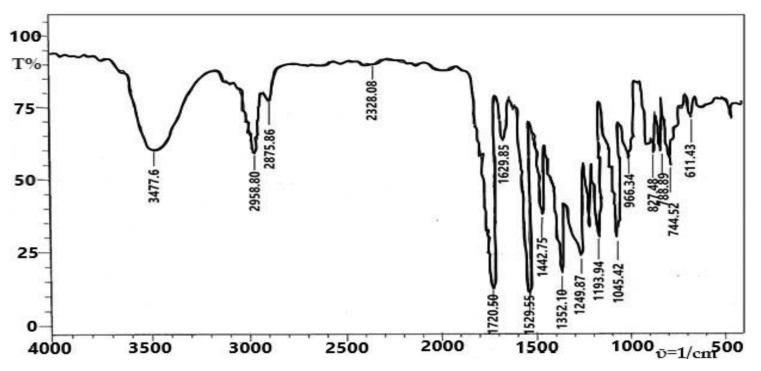
FT-IR experimental spectrum of methyl 2-((2-nitrophenyl)(hydroxyl)methyl) acrylate (MNHA).

**Figure 3:**
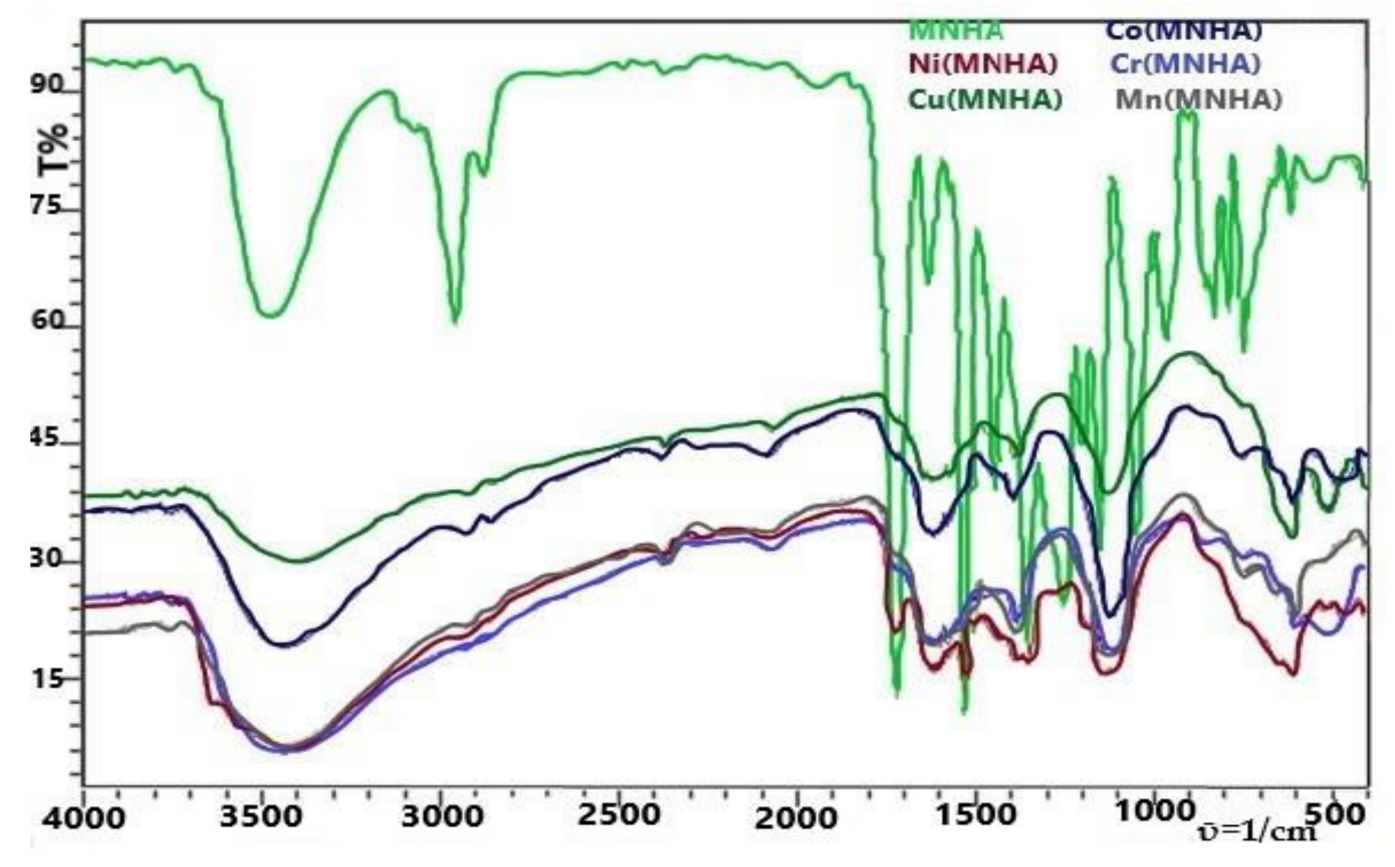
FT-IR overlay patterns of compounds **3–8**.

Detailed information of the experimental and theoretical FT-IR vibrational frequencies of the ligand, MNHA, and its corresponding metal complexes (compounds **3**–**8**) are given in Table 3. However, the appropriate amount of chosen catalyst as well as the solvent often had a significant effect on the stability, rate, and efficiency of the MBH reactions. Using THF as the solvent and DABCO as the catalyst, the MBH adducts appeared form as major products in moderate to good yield, when the reaction set-up was maintained under continuous stirring for up to 24 h. The confirmation of the stability of the adduct (MNHA) was achieved via spectral analysis, which showed that the adduct was not dimerized. Additionally, the ^1^H NMR and mass spectra indicated good agreement with our findings [52,53]; hence, the purity of the final product (MNHA) synthesized was proven using this procedure. Moreover, the obtained theoretical values closely resembled the experimental values (Table 3), and hence, suggested a good agreement between the proposed and actual molecular structure [54] of the MNHA ligand (Figure 1). FT-IR values for compound **3** (Figure 1) showed the presence of -OH functional groups with a peak nearly at 3480–3380 cm^−1^, as was indicated in the theoretical spectrum of the ligand (Figure 2). In contrast, in FT-IR spectra of compounds **4**–**8**, broad bands were observed in the range of 3500–3300 cm^−1^, which were attributable to -OH because of the presence of moisture or coordinated water molecules in the samples of all of the complexes [30,54,55]. Moreover, NO_2_ peaks in the ligand appeared at 1529 cm^−1^, 1552 cm^−1^, but were found to be entirely absent after complex formation. Insignificant changes in the peak positions of C-H sp^2^ and sp^3^ bonds of complexes were noted. The frequencies of the C=C (aliphatic) bond and the C=C (aromatic) bond also did not change, signifying non-involvement of these sites in complex formation [56,57]. A long pointed sharp peak of C=O in the ligand at 1720 cm^−1^ was observed to only slightly shift towards the lower position (ῡ = 1716), but more importantly, also weakened in intensity in the complex (compound **4**). This suggests that upon coordination of this site to metal ions, the sharp peak transformed into a peak of either weak or almost negligible intensity at 1716 cm^−1^. This fact strongly confirmed the coordination of metal ions through the carbonyl functional group [58]. Additionally, the -OH exhibited peaks at 3450–3300 cm^−1^ (broad) and =CH groups at 2931 cm^−1^ with the minor changes, while the NO_2_ group gave a very weak peak at 1525 cm^−1^. Furthermore, the peaks at 790 cm^−1^, 609 cm^−1^, and 464 cm^−1^ were assigned to M-OH, M-O, and M-NO_2_ groups (M=Ni^2+^), respectively. We instantly concluded that C=C (1610 cm^−1^) did not participate in complexation, as no significant shift in its peak position was observed (Figure 3), although a little modification in the observed values may be due to the neighboring functional groups, as evidenced from their sharp pointed peaks at exact frequencies [44,59]. Likewise, the spectrum of the copper complex (compound **5**) showed a broad band of the -OH functional group, with little change in the position of the region 3480–3380 cm^−1^ as compared to compound **3** (Table 3). Close inspection of the IR spectrum of compound **5** revealed the disappearance of some peaks, which were justified by the appearance of additional peaks at 782 cm^−1^, 607 cm^−1^, and 472 cm^−1^ because of the existence of Cu-OH, Cu-O, and Cu-NO_2_ bonds [36]. Here, in this compound, strong interaction was observed by the NO_2_ functional group of the ligand. This is justified, as two sharp peaks assigned to the NO_2_ functional group of the ligand at 1529 cm^−1^ and 1552 cm^−1^ disappeared in the complex, which is a solid reason for the coordination of the ligand from the nitro group as well. By keeping the analytical, spectral, and physical findings in mind, the prepared complex was assigned the structure presented in Scheme 2, and formulated as [Cu(MNLA)_2_] (compound **5**).

In the FT-IR spectrum of the cobalt complex (compound **6**), a broad signal in the form of a broad peak ranging from 3500–3350 cm^−1^ was observed, providing the information of the -OH group. The signal was at a similar position as that in the ligand spectrum, but was in the form of a moderately broad peak. A weak but clear signal appeared at 1651 cm^−1^ that initially was at 1720 cm^−1^ in the ligand; this is possible because of the carbonyl group. This was a significant frequency shift compared to the ligand spectrum, not only in position, but also in intensity. This kind of shift confirmed the coordination of the ligand’s carbonyl site to the Co^3+^ ion [60,61]. Another significant difference in the IR spectrum of complex was a minor shift to a greater ῡ in the IR signal of C-O (of ester), i.e., the ligand C-O frequency shift from 1249 to 1390 (C-O of the complex) may have arisen due to environmental effects during the coordination [62]. Moreover, an exclusive signal that was attributable to Co-OH at 748 cm^−1^ and Co-O at 651 cm^−1^ appeared, while another noticeable variation in Co-NO_2_ frequency from 607 cm^−1^ was also observed [63]. When the IR spectrum of compound 7 was studied, the significant changes ensured coordination of the ligand through the oxygen atom of the C=O group to the Cr^3+^ [64]. The insignificant shift in position means that the C=C functional group did not contribute to coordination with the central metal ion and the aromatic ring C=C that showed frequencies at almost the same positions [29,46]. In its respective complex formation, the ligand aromatic pi bonds also suggested non-coordinating behavior of the C=C of the aromatic ring [49]. An additional peak observed at 514 cm^−1^ usually appears due to Cr-NO_2_ bonding, and hence proves the involvement of -NO_2_ in coordinate bond formation to the metal ion. A few more peaks were also observed at 842 cm^−1^ and 603 cm^−1^, which were allocated to Cr-OH and Cr-O coordination, respectively.

In compound **8**, disappearance of the sharp signal of the carbonyl group provided a clue of its involvement in the coordination. The same kind of disappearance of peaks has also been explained in the above metal complexes, so it likewise confirms the coordination of the ligand’s carbonyl site to the Mn^2+^ metal ion [51]. In addition, a significantly small peak at 750 cm^−1^ was in accordance with the presence of M-OH, and that at 611 cm^−1^ was attributed to M-O; meanwhile, the peak at 509 cm^−1^ was assigned to M-NO_2_ (M=Mn^2+^). Moreover, the peaks of the -NO_2_ group, originally present in the ligand, were found to be missing in compound **8**. FT-IR overlay patterns for compounds **3**–**8** are shown in Figure 3.

### 4.4. Mass Spectrometry

Mass spectroscopy based on *m/z* 188 and 39.5% yielded a total of 373 ions and confirmed the successful synthesis of compound **3** through Scheme 1. The 77 finest ions with the lowest % are because of benzene (C_6_H_6_^+^), but at the lowest m/e (13%). Other small fragments were also observed, which are definitely because of the shifting of nitro group, resulting in resonating structures [26]. Furthermore, these fragments had very low % of m/e. Moreover, at 100% of m/e, there was a mass of 188 that conformed to the presence of C_11_H_9_O_4_N^+^. The fragment having a mass of 191 exactly matched with C_10_H_12_O_2_^+^ at 25%, while the ionic fragmentation (220) was due to C_11_H_13_O_4_N^+^ of *m/z* 71%. The aforementioned mass was due to the molecular ion peak without the alcoholic -OH group in the structure, but we also observed mass peaks of 238 in exceptionally low percentages of *m/z* ratio, conforming to the presence of M+1. The estimated mass fragments of transition metal complexes matched with their molecular masses. Meanwhile, the experimental mass spectrum could not be achieved, because the metal complexes were insoluble in any solvent other than DMSO. Moreover, the fragmentation peaks of all the complexes (compounds **4**–**8**) were without water molecules, justifying their molecular masses [56,65]. The first fragment of each complex resulted because of a lack of a methoxy (OCH_3_) group. The other fragments with certain masses were due to the loss of C_2_H_3_O_2_^+^ (59), C_4_H_5_O_2_^+^ (85.15), and C_5_H_7_O_3_^+^ (116.04) fractions at higher percentages, comparatively. The theoretical molecular ion peaks of each metal complex were in good agreement, confirming compounds **4**–**8** that were obtained through Scheme 2.

### 4.5. ^1^H NMR Spectroscopy of Compound ***3*** (MNHA)

The ligand (MNHA) in its ^1^H NMR spectrum presented five prominent signals due to -OH, -OCH_3_, =CH_2_, and -CH at 3.419, 3.707, 5.705, 6.175, and 6.342 ppm, respectively. Additionally, aromatic ^1^H gave one signal at 7.426 ppm, and a second signal (^1^H) at 7.607 ppm, which is noticeable as a triplet. As a result of the aromatic ring protons [66], very clear signals of two doublets appeared at 7.737 and 7.915 ppm, respectively, in the spectrum of ^1^H NMR.

### 4.6. Geometrical Optimization

**Figure 4:**
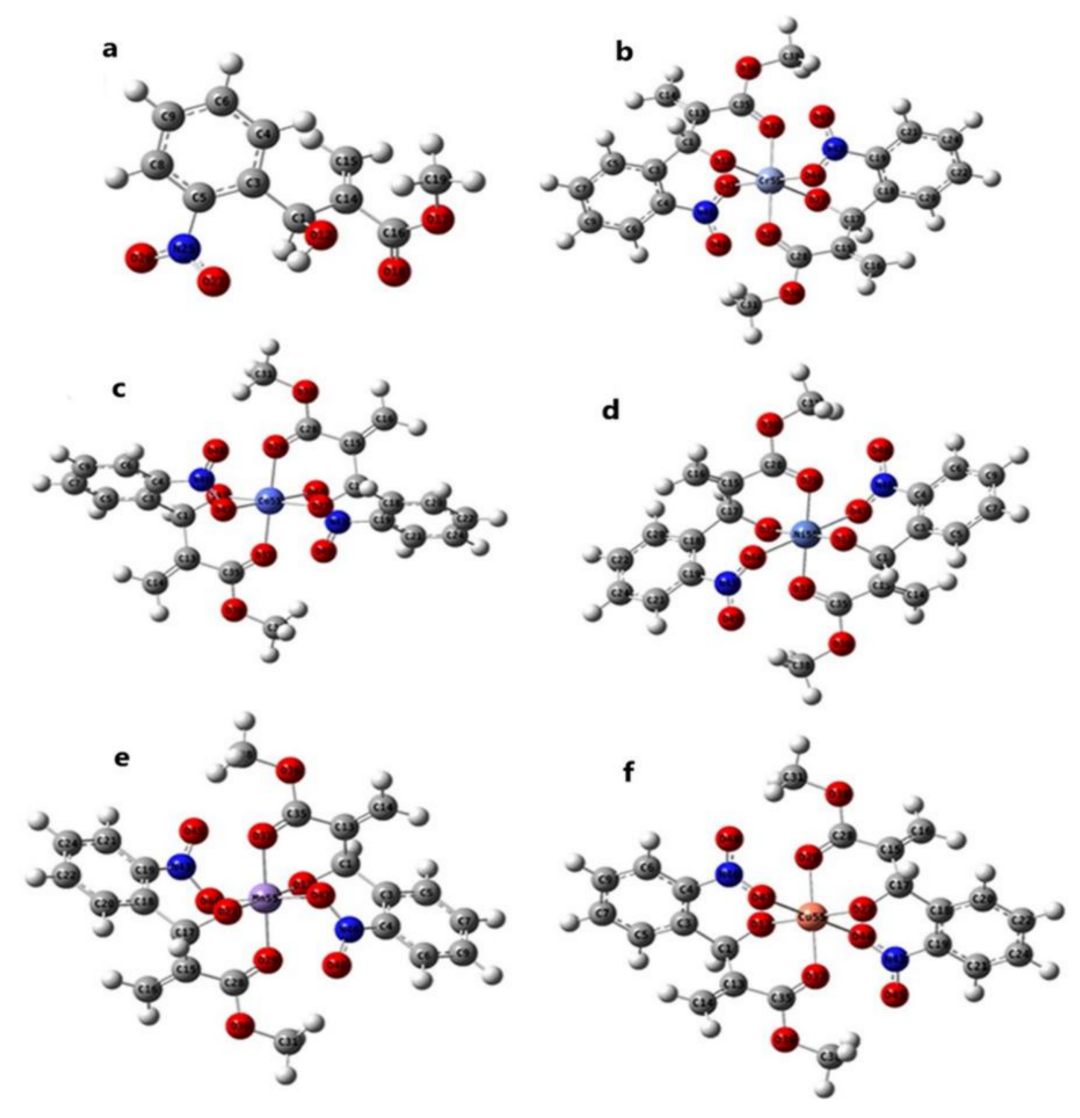
Theoretically optimized geometries of compounds **3**–**8**. (**a**) [(MNHA)], (**b**) [Cr(MNHA)_2_]^+^, (**c**) [Co(MNHA)_2_]^+^, (**d**) [Ni(MNHA)_2_], (**e**) [Mn(MNHA)_2_], and (**f**) [Cu(MNHA)_2_]. The stable geometries confirmed each metal coordinated octahedrally with two tridentate ligand molecules.

For geometrical optimization as well as frequency calculations, the B3LYP and 6-311++ G (d.p) basis set was utilized. Subsequently, the synthesized ligand (MNHA) was found to be in good agreement with the observed FT-IR spectrum, after computational resolution. The novel compounds were structurally analyzed via physical, analytical, and spectral techniques. Meanwhile, the semi-empirical PM-6 method was used for geometrical optimization of each metal complex (compounds **4**–**8**). The correlation between the computational as well as experimental investigations gave reasonable information on the geometrical shapes and binding confirmations [67,68]. The resultant optimized geometries of these novel compounds, along with the ligand, are presented in Figure 4.

### 4.7. X-ray Powder Diffraction

**Figure 5:**
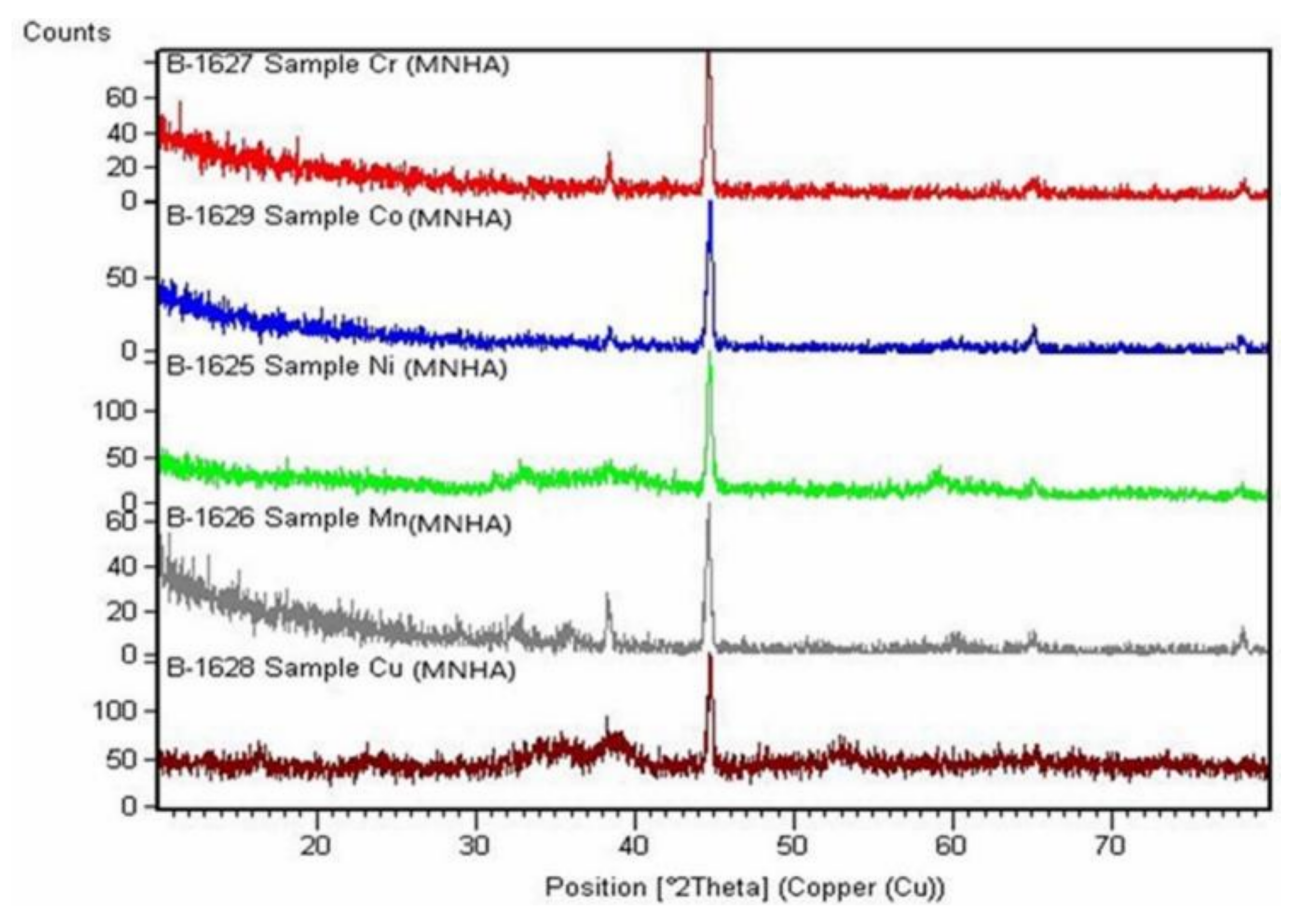
XRD powder diffraction patterns of compounds **4**–**8**.

The crystalline nature of metal complexes (compounds **4**–**8**) was determined through the X-ray powder diffraction studies. The single crystals of these metallodrugs could not be achieved, even after many attempts. However, the definite size of crystals obtained after calculation of parameters found via powder-XRD confirmed that these were in crystalline form [69]. The powder-XRD method is one of the best tools to optimize the crystal class, along with the size of compounds [44,57]. The diffractogram patterns that were obtained with the sharply pointed peaks are given in Figure 5. The sharp pointed peaks of XRD patterns are an excellent indication of the crystalline nature of the formed compounds. It was observed that these patterns were different from each other, and hence, were attributed to the well-defined unique crystal structure in all forms. The observed variation may be because of moisture [70], as well as different binding modes of the ligand in the coordination sphere [71,72]. Furthermore, the indication of a crystalline nature of all of the complexes, and the dissimilarity from each other owing to a distinct distorted crystalline structure formation, are the main achievements of this study. In addition, all of the complexes possessed non-identical unit cell numbers as well as RIRs (radius intensity ratio), because they belonged to different and distinctive crystal classes [73,74]. The resultant XRD characteristics, such as volume, density, RIR, a, b, c, α, β, γ (lattice parameters), and possible crystal class, are given in Table 4. The nickel complex (compound **4**) is hexagonal, with a P6322 (182) space group, and the unit cell number is 2. In the case of the copper complex (compound **5**), the crystal class was identified as orthorhombic. As a result of the non-identical characteristics of crystalline features [30], it also possessed the dissimilar space group Bmm2 (38), while its Z value was 2.

The chromium, cobalt, and manganese metal complexes (compounds **6**–**8**) were found to be in the same crystalline system of a cubic nature. However, all of these three complexes had entirely different characteristics related to their physical behaviors [57,71]. The space group of the chromium metal complex (compound **7**) was Pm-3n (223), the cobalt metal complex (compound **6**) was of Fd-3m (227), and the manganese metal complex possessed a P6322 (182) space group. The complexes of chromium, cobalt, and manganese (compounds **6**–**8**) had 2, 8, and 4 Z values, respectively. The indexing associated to clarification of their crystal structures and peak intensities along with diffraction angle (2𝜃) is given in Table 5. For the stability confirmation, the size of grains of amorphous compounds is very significant. In this subject, XRD-powder diffraction is a powerful technique to measure the size of microcrystals present in amorphous samples [75]. Scherer’s equation is frequently used for the calculation of grain size [76], and is formulated as follows:

(1) $$S=\frac{\;\lambda m}{\beta \;Cos\theta }$$

In Equation (1), **m** is a constant value (0.94), **λ** shows the wavelength of the X-rays, ***θ*** shows the angle of Bragg’s diffraction, while ***β*** shows FWHMI (full width of half maximum intensity). In the present study, the average grain size (***S***) was calculated from the highest peak intensity in units of nm (Table 5). However, the nano-crystals were single phase or multiphase polycrystalline solids with a crystal size of only a few nm (usually less than 100 nm). Herein, the calculated size was 4.5–10 nm (Table 4) of all of the synthesized metal compounds; therefore, they belong to a nano-crystal class of amorphous compounds [74].

### 4.8. Antibacterial Assay

**Figure 6:**
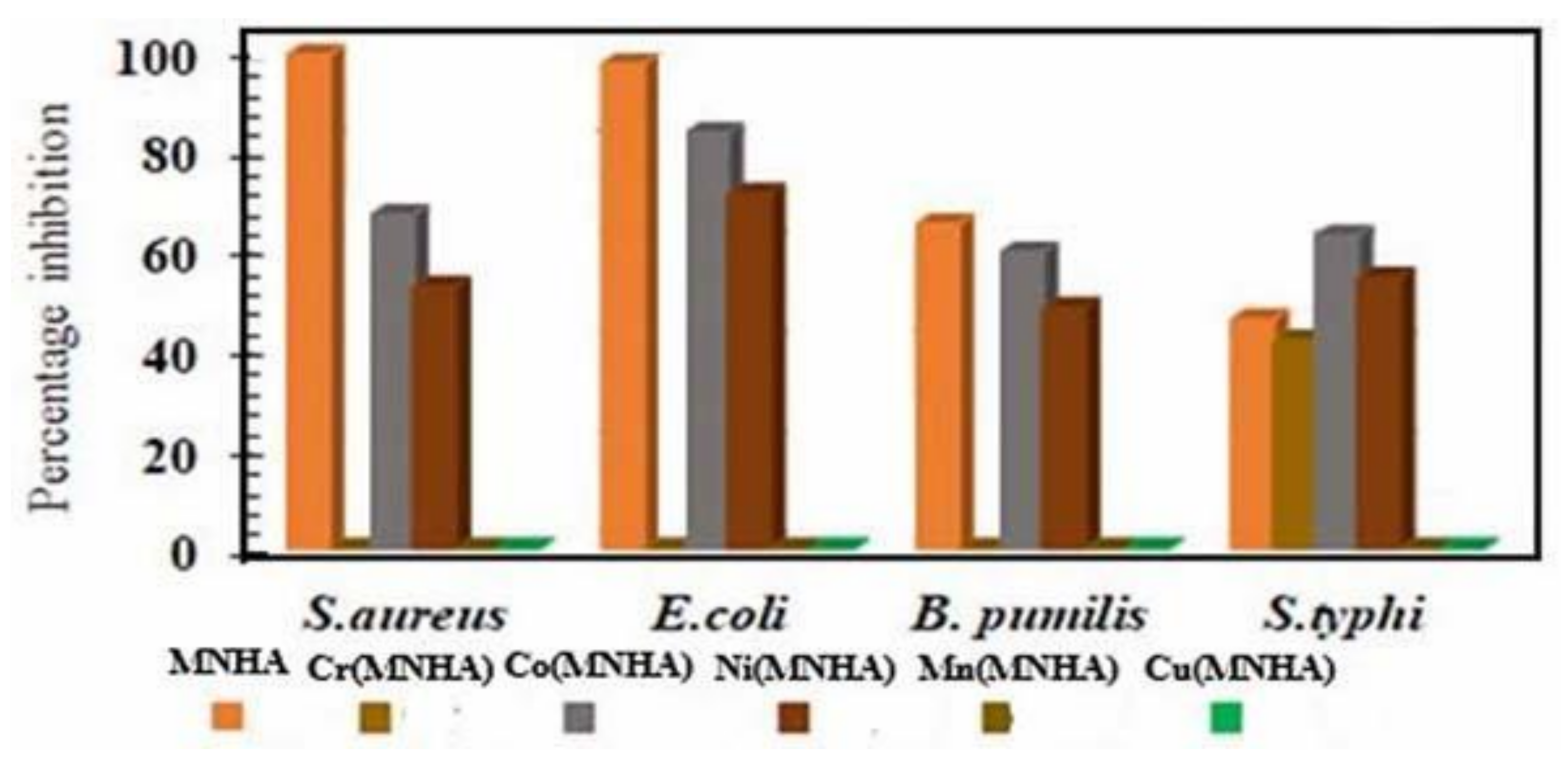
Comparative histograms of compounds **3**–**8** (*S.typhi, E.coli* = Gram-negative bacteria and *B.subtilis, S.aureus* = Gram-positive bacteria) in terms of % inhibition zone.

Table 6 shows the results for antibacterial activity of compounds **3**–**8**, monitored through the agar disc diffusion technique [36,70]; the results are presented in Table 6 and Figure 6. The activity index was evaluated through the relationship given in Equation (2):

(2) $$\left(\%\right)\mathrm{Activity}\;\mathrm{Index}\;=\;\mathrm{IZ}\;\mathrm{of}\;\mathrm{compound}/\;\mathrm{IZ}\;\mathrm{of}\;a\;\mathrm{standard}\;\mathrm{drug}\;\times 100$$

where, ‘**IZ**’ is inhibition zone in mm. The ligand (MNHA) proved to be an excellent antibacterial agent against all of the four chosen bacterial strains, with 98.6% and 96.8% activity for *S. aureus* and *E. coli*, respectively. Some contrasting results for the metal complexes were obtained, which showed average to moderate antibacterial potential of these compounds, with even non-activity of many of these complexes. This is a consequence of chelation of the organic ligand to the central metal ion, which decreased its antibacterial potential [77,78].

### 4.9. Antioxidant Activity

**Figure 7:**
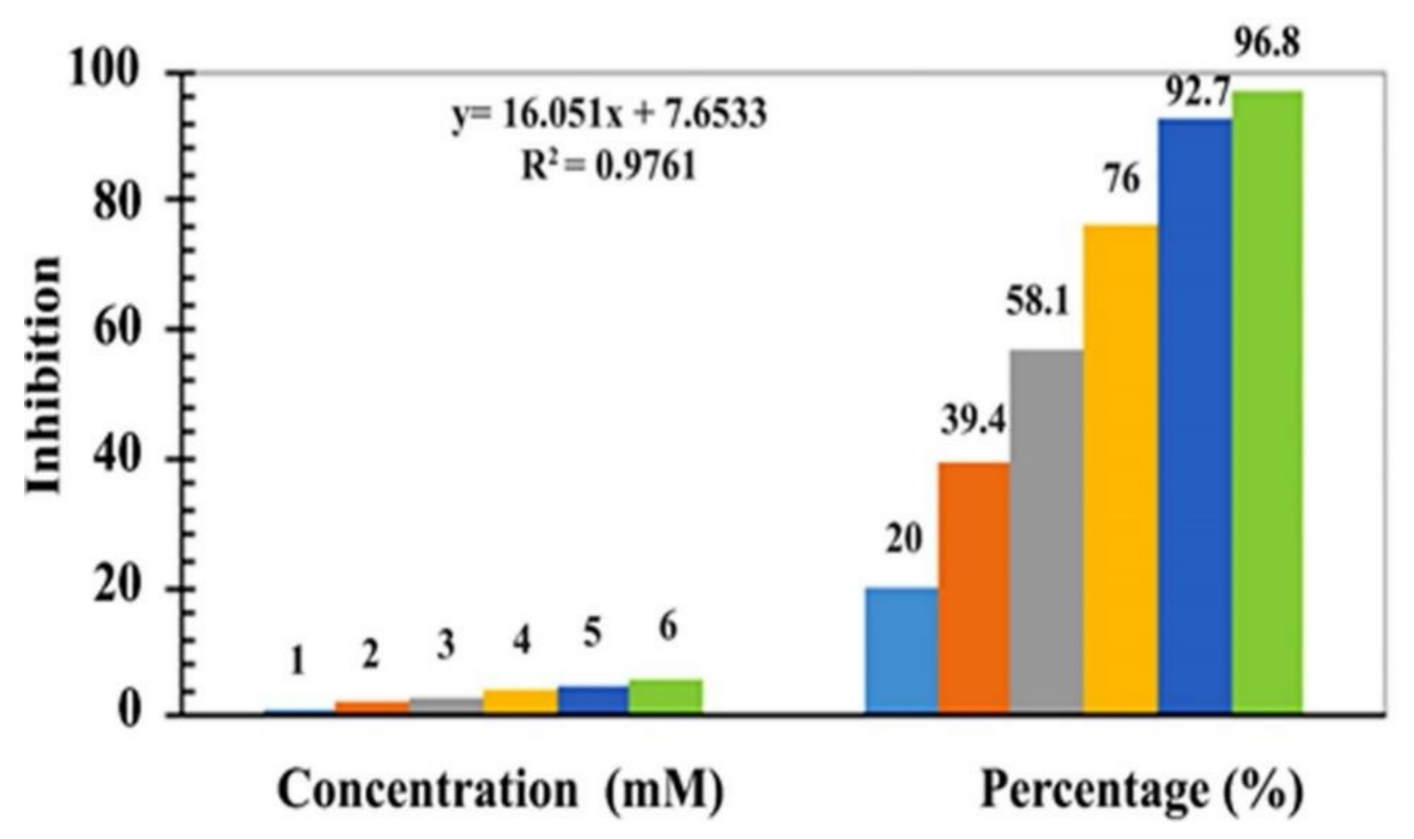
Graphical representation of antioxidant activities of ascorbic acid (standard).

**Figure 8:**
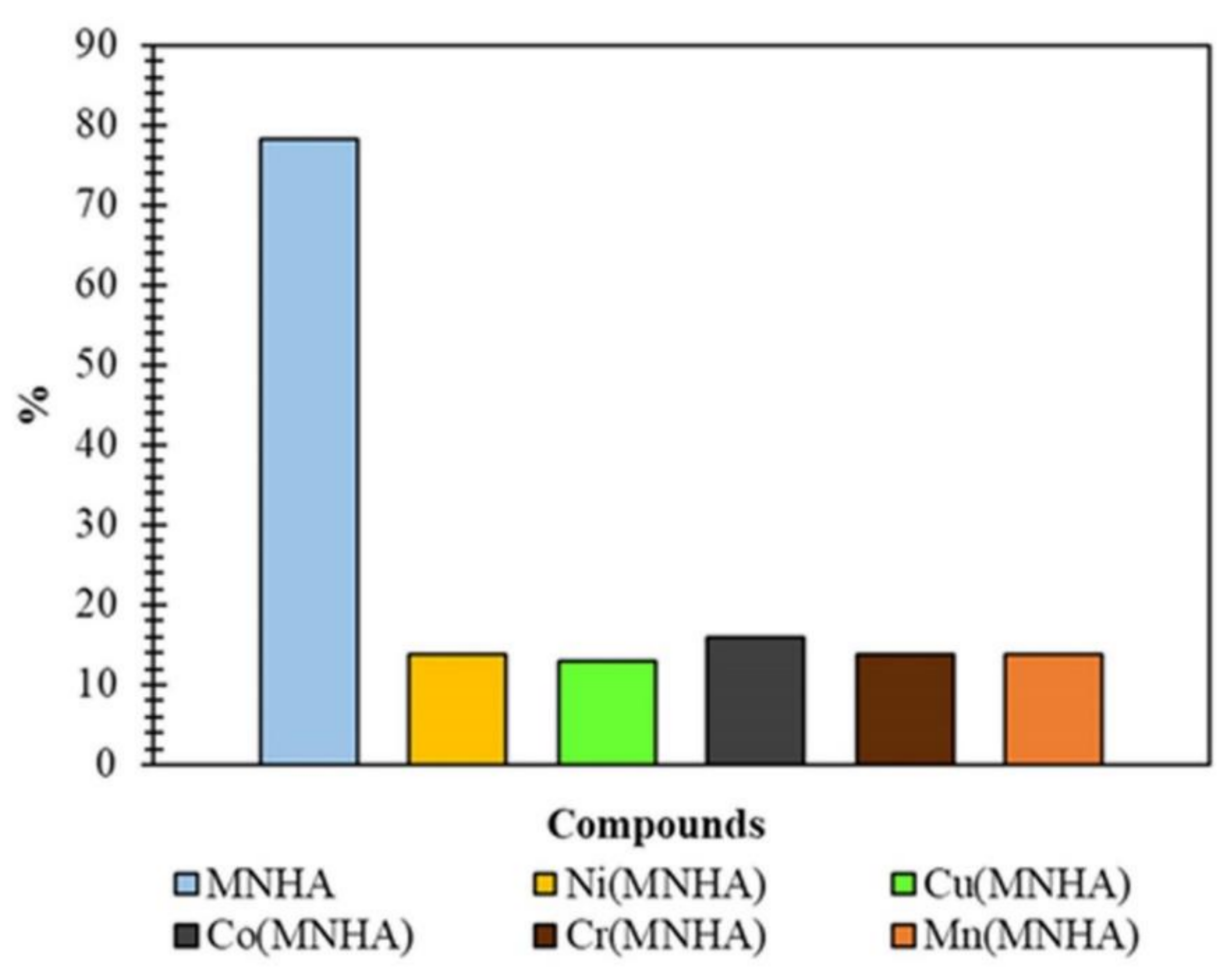
Graphical representation of antioxidant activities of MNHA and its corresponding metal complexes.

The radical scavenging activity (%) was determined through the following relation:

(%) DPPH scavenging efficiency = Ac − As/Ac × 100(3)

where ‘**Ac**’ is the absorbance of control (radical), while, ‘**As**’ is absorbance of standard. Figure 7 and Figure 8 present activity of the standard (ascorbic acid) and compounds **3**–**8**, respectively. The multifunctional organic aromatic compounds were extremely active antioxidants [3,37]. Therefore, the prepared adduct MNHA (compound **3**) presented an excellent antioxidant potential due to the presence of both alcoholic and ester functional groups, along with other moieties in its structure. On the other hand, all the novel metal complexes showed little but positive potential as free radical scavengers [58,79].

### 4.10. Antifungal Activity

**Figure 9:**
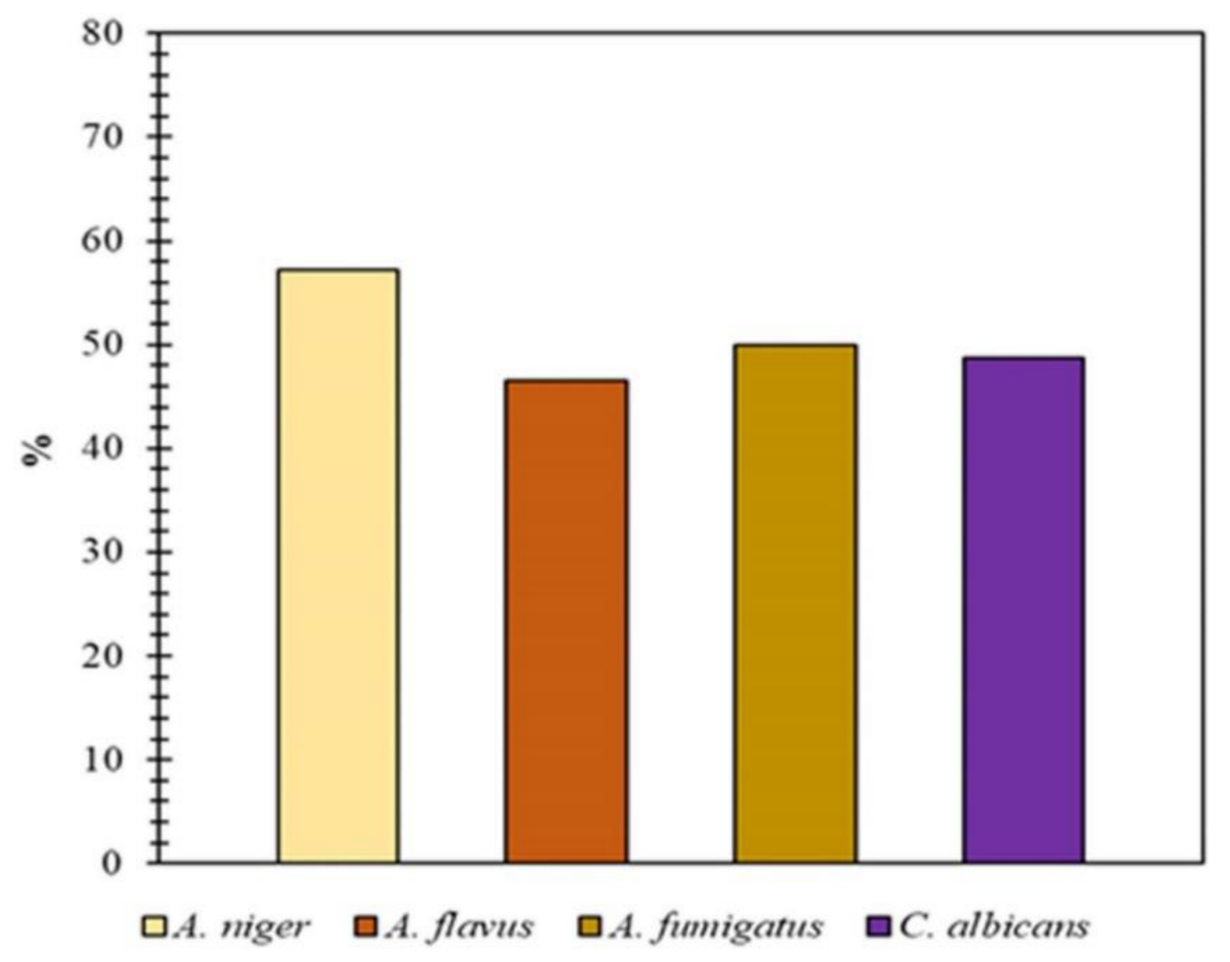
Antifungal activity of MNHA. Inhibition of mycelial growth (%) = Δd/dc × 100 (Δd = dc − dt); dc = average diameter of fungal colony in negative the control, while dt = average diameter of fungal colony in experimental plates.

Figure 9 presents the % antifungal activities of compounds **3**–**8,** tested against four fungal strains (*A. Niger, A. flavus, A. fumigatus, C. albicans*) via a quantitative agar well diffusion method [80]. It was observed that the MBH adduct (compound **3**) acted exceptionally against the fungal strains [9], although against four selected fungal strains, the metal complexes were totally inactive. This may have been a consequence of nitro group participation in the coordination towards central metal ions [78]. When the nitro group in the ligand (compound **3**) was free, it could provide surplus active bonding against fungal pathogens [81] and act as an excellent antifungal agent.

### 4.11. Molecular Docking of Compounds ***3***–***8***

**Figure 10:**
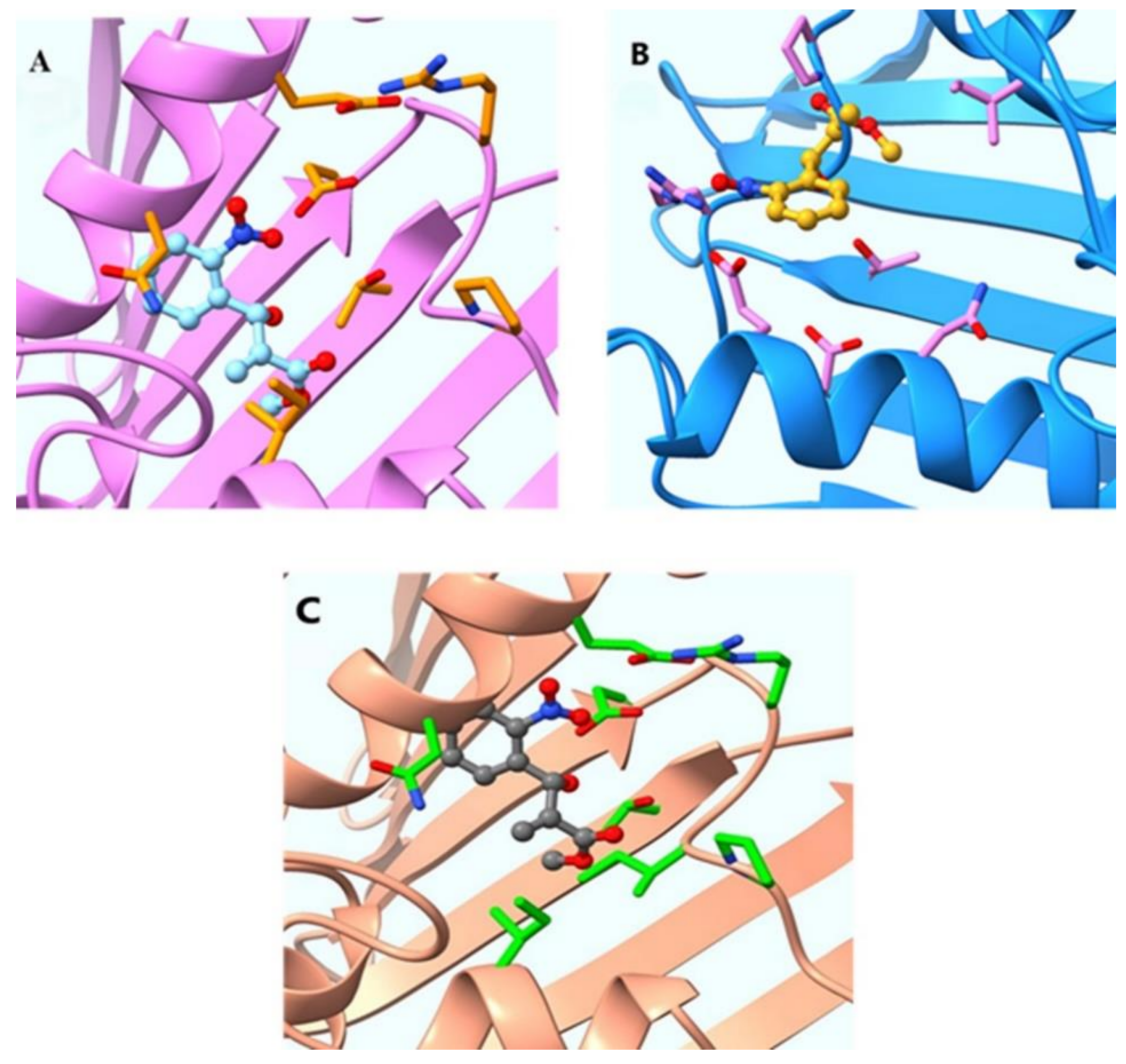
The binding conformations of the MNHA ligand against (**A**) *S. typhi*, (**B**) *S. aureaus*, and (**C**) *E. coli*.

**Figure 11:**
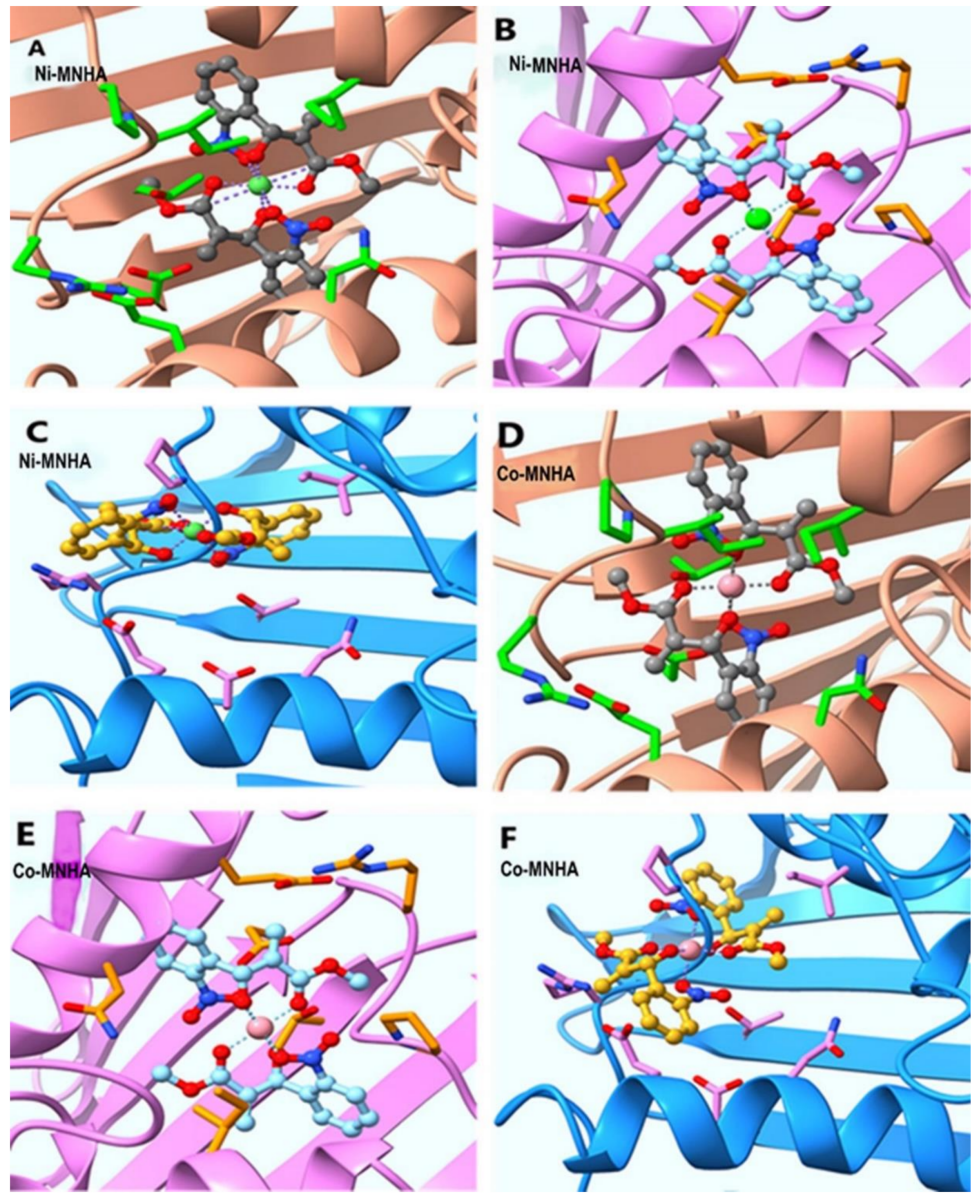
The binding conformations of ligand (MNHA) metal complexes against (**A**,**D**) *E. coli*, (**B**,**E**) *S. typhi*, and (**C**,**F**) *S. aureaus*.

The biological potential of the prepared ligand (MNHA), along with the complexes, were further checked via their binding ability towards the proteins of *E. coli, S. aureus*, and *S. typhi* through molecular docking studies. Results from docking studies provide concrete support to the observed antibacterial activities of the ligand and its metal complexes. In Figure 10A–C, the panels are related to the MBH adduct ligand (MNHA) binding with the CDP-D-glucose 4,6-dehydratase from *S. typhi*, with the ATP binding site of *S. aureus* DNA-gyrase, and with *E. coli* DNA gyrase B. The substrate binding site residues of *S. typhi* dehydratase from PDB-ID: 1WVG were S134, D135, K136, Y159, N197, and R208, which were bound with CDP-D-xylose. The ATPase binding site residues of *S. aureus* DNA-gyrase from PDB-ID: 4UR0 were S55, A64, N65, V79, D89, T164, and T173, which were found engaged with novobiocin. The ATPase binding site residues of *E. coli* DNA gyrase B from PDB-ID: 1AJ6 were N46, D73, R76, V120, R136, and T165, which were originally unavailable for novobiocin. In Figure 11A–C, the panels are related to the metal complex Ni(MNHA) binding with *E. coli* DNA gyrase B, with CDP-D-glucose 4,6-dehydratase from *S. typhi*, and with the ATP binding site of *S. aureus* DNA-gyrase, respectively. In Figure 11D–F, the panels are related to the metal complex (Co-MNHA) binding with *E. coli* DNA gyrase B, CDP-D-glucose 4,6-dehydratase from *S. typhi*, and the ATP binding site of *S. aureus* DNA-gyrase, respectively. Ligand (MNHA), Co(MNHA), and Ni(MNHA) were found to be well-occupied inside the active site of three (*E. coli, S. typhi,* and *S. aureus*) antibacterial protein targets.

## 5. Conclusions

In this study, methyl 2-((2-nitrophenyl)(hydroxy)methyl) acrylate (compound **3**), an aromatic MBH adduct (MNHA), was efficiently synthesized using a well-established MBH reaction method (Scheme 1). The adduct further served as a ligand for the synthesis of novel transition metal complexes (compounds **4**–**8**) (Scheme 2). The physical, analytical, and spectral analyses confirmed the formation of compounds **3**–**8** as expected. Additionally, there was good agreement in the observed and computed, DFT B3LYP method, and the generated FT-IR spectrum of compound **3** supported the successful synthesis. Semi-empirical PM6, 6-311++ G (d,p) basis set method was applied, and proved to be a powerful tool for geometrical optimization of these synthesized novel species (metal complexes). Computational investigations of all of the synthesized compounds provided reasonable geometric shapes and binding conformations, which were well correlated with the experimental results. These studies collectively endorsed the molecular and structural formulas of the novel metal complexes. XRD powder diffraction revealed that both Ni^2+^ and Cu^2+^ metal complexes with MNHA (compound **3**) belonged to the hexagonal and orthorhombic crystal classes, respectively. Meanwhile, the remaining metal complexes (Cr^3+^, Co^3+^, Mn^2+^) were cubic crystalline in nature. The proposed molecular and structural formulas that were obtained by the combined results came out to be a 2:1 ligand to metal ratio in these complexes. Moreover, the collective investigations showed that MNHA has acted as a tridentate ligand because of presence of -OH, -C=O, and -NO_2_ functional groups. Furthermore, the compounds also possess biological potential, as evidenced from their antibacterial, antifungal, and antioxidant activities. Through molecular docking studies, the antibacterial potentiality of the prepared ligand as well as the complexes (compound **3**–**8**) was successfully tested through their binding ability towards the proteins of *E. coli, S. aureus*, and *S. typhi*. Compound **3** was concluded to be s the most effective drug among these products for a range of fungal pathogens, in comparison to the resultant metal complexes. The antibacterial potential of some of the complexes suggests their use as potent antibiotics in drugs. The inclusive biological findings of the current study could be beneficial in improving the efficiency of some therapeutic drugs.

## Figures and Tables

**Table 1:** Physical and analytical data of compounds **3**–**8**.

Compound	Molecular Formula	M. Wt (g/mol)	Color	M.P(°C)	Analysis (%) Calc. (Found)			
					C	H	N	O
MNHA (3)	C_11_H_11_NO_5_	237.06	Orange brown	124.3–126.5	55.70 (55.20)	4.67 (4.56)	5.90 (6.1)	33.72 (32.98)
Ni(MNHA) (4)	C_22_H_2_0N_2_NiO_10_	531.10	Penny brown	>300	49.56 (50.20)	3.80 (4.35)	5.27 (5.9)	30.13 (31.05)
Cu(MNHA) (5)	C_22_H_20_CuN_2_O_10_	535.95	Shamrock green	>300	49.30 (50.12)	3.12 (4.54)	5.23 (5.38)	29.85 (28.96)
Co(MNHA) (6)	C_22_H_20_CoN_2_O_10_^+^	531.33	Dark gray	>300	49.73 (49.54)	3.79 (4.05)	5.27 (5.03)	30.11 (30.84)
Cr(MNHA) (7)	C_22_H_20_CrN_2_O_10_^+^	524.05	Caramel brown	>300	50.39 (50.89)	3.84 (4.16)	5.34 (5.07)	30.51 (30.10)
Mn(MNHA) (8)	C_22_H_20_MnN_2_O_10_	527.05	Amber brown	>300	50.11 (50.65)	3.82 (3.53)	5.31 (5.97)	30.34 (30.16)

**Table 2:** Investigated UV–Vis spectroscopic data for the MBH adduct ligand (MNHA) and its formed complexes.

Sample (s)	Concentration 1 × 10^−3^ (M)	λmax (nm)	ɛ	Absorbance
			(mol^−1^cm^−1^L)	(A)
MNHA	1.051	250	676	0.71
		266	770.69	0.81
		275	808.76	0.85
		300	57.088	0.06
Ni(MNHA)	1.049	264	2694	0.132
		283	108.67	0.114
		370	74.36	0.078
Cu(MNHA)	1.047	266	209.17	0.219
		290	169.05	0.177
		371	108.88	0.114
Co(MNHA)	1.05	268	42.86	0.045
		308	60.95	0.064
		371	115.23	0.121
Cr(MNHA)	1.049	265	27.65	0.029
		308	20.97	0.022
		371	65.78	0.069
Mn(MNHA)	1.048	268	432.25	0.453
		286	317.75	0.333
		366	110.69	0.116

All compounds were dissolved in DMSO to observe the UV–visible spectrum.

**Table 3:** Characteristic FT-IR bands (ῡ = cm^−1^) of compounds **3–8**.

Functional Group	(MNHA) Exp.	(MNHA) Calc.	Ni (MNHA)	Cu (MNHA)	Co( MNHA)	Cr (MNHA)	Mn (MNHA)
ῡ (-OH)	3477	3659	3450–3300	3480–3380	3500–3350	3500–3400	3450–3350
ῡ (=C-H)	2958	3136	2931	2926	2929	2950	2926
ῡ (sp^3^ C-H stretch)	2875	3037	2860	2856	2858	2800	2856
ῡ (ester C=O)	1720	1677, 1697	1716	-	-	-	-
ῡ (aromatic C=C)	1629	1612	1610	1622	1597	1625	1610
ῡ (-N=O)	1529, 1552	1434	1525	-	1573	1527	1560
ῡ (-CH_3_)	1442	1483, 1521	1431	-	-	-	-
ῡ (C-O)	1249	1235	1384	1388	1390	1390	1388
ῡ (C-OCH_3_)	1149	1159	1141	1114	1114	1177	1128
ῡ (-OH bend)	1045	980, 438	1111	-	-	1107	-
ῡ (=C-H aromatic)	744	748	651	-	-	750	-
ῡ (M-O)	-		609	607	651	603	611
ῡ (M-OH)	-		790	782	748	842	750
ῡ (M-NO_2_)	-		464	472	607	514	509

Observed changes in the frequencies of specified functional groups.

**Table 4:** Observed refinement parameters and crystallographic data for complexes (compounds **4**–**8**).

Compound	S. Group (G. No.)	Volume (10^−6^ pm^3^)	Density (g/cm^3^)	Unit Cell Dimensions (Å)	Crystal Class	No. of Unit Cells (Z)	Reference Intensity Ratio (RIR)	S (nm)
Ni(MNHA)	P6322 (182)	79.31	7.96	a = b ≠ c α = β = 90° γ = 120°	Hexagonal	2	6.40	9.9876
Cu(MNHA)	Bmm2 (38)	307.56	5.66	a ≠ b ≠ c α = β = γ = 90°	Orthorhombic	2	4.97	6.9064
Co(MNHA)	Fd-3m (227)	528.30	6.05	a = b = c α = β = γ = 90°	Cubic	8	5.32	4.6041
Cr(MNHA)	Pm-3n (223)	93.82	6.09	a = b = c α = β = γ = 90°	Cubic	2	6.39	5.9128
Mn(MNHA)	Fm-3n (225)	42.51	8.58	a = b = c α = β = γ = 90°	Cubic	4	7.53	5.9015

**Table 5:** Summary of data collection (crystal lattice records) and modified parameters for compounds **4**–**8**.

Complex Compound	Miller Indices			d [Å]	2° 𝜃	Intensity (%)
	H	K	L			
[Ni(MNHA)_2_]	1	1	0	2.308	38.993	18.8
	0	0	2	2.149	42.01	23.5
	1	1	1	2.0333	44.522	100
	1	1	2	1.572	58.615	14.7
[Cu(MNHA)_2_]	−1	1	1	2.524	35.539	100
	1	1	1	2.323	38.731	100
	2	0	0	2.311	38.941	100
	0	0	2	2.431	35.438	60
[Co(MNHA)_2_]Cl	2	2	0	2.86	31.249	40
	3	1	1	2.438	36.837	100
	5	1	1	1.555	59.35	35
	4	4	0	1.4293	65.222	45
[Cr(MNHA)_2_]Cl	1	1	0	3.213	27.742	27.2
	2	0	0	2.272	39.637	16
	2	1	0	2.0321	45.551	100
	2	1	1	1.855	49.069	28.6
[Mn(MNHA)_2_]	0	1	1	3.08	28.967	40
	1	0	3	2.76	32.412	60
	2	1	0	2.49	36.041	80
	1	2	4	2.03	44.6	100

On the basis of highest intensity (%) of sharp peak, each set of indices was chosen.

**Table 6:** Antibacterial assay of compounds **3**–**8**.

Compound	*S. aureus* (%)	*E. coli * (%)	*B. pumilis* (%)	*S.typhi* (%)
MNHA	98.6	96.8	64.8	45.8
Ni(MNHA)	52.3	70.8	48.1	54.1
Cu(MNHA)	-	-	-	-
Co(MNHA)	66.6	83.0	59.2	62.5
Cr(MNHA)	-	-	-	41.6
Mn(MNHA)	-	-	-	-

Inhibition zone diameter in mm of synthesized compounds against pathological strains based on an agar well diffusion method at 1 mg mL^−1^. The experiment was carried out in triplicate, and the average zone of inhibition was calculated. (-) = non-active inhibition zone.

## Data Availability

Not applicable.
